# Supplementary figures and images for: Alpha-centractin is a novel substrate of SETD3 methyltransferase in vitro
Source: PeerJ. 2025 Oct 20;13:e20195. doi: 10.7717/peerj.20195 (PMC12548662; doi:10.7717/peerj.20195)

## Supplementary Figure 1.

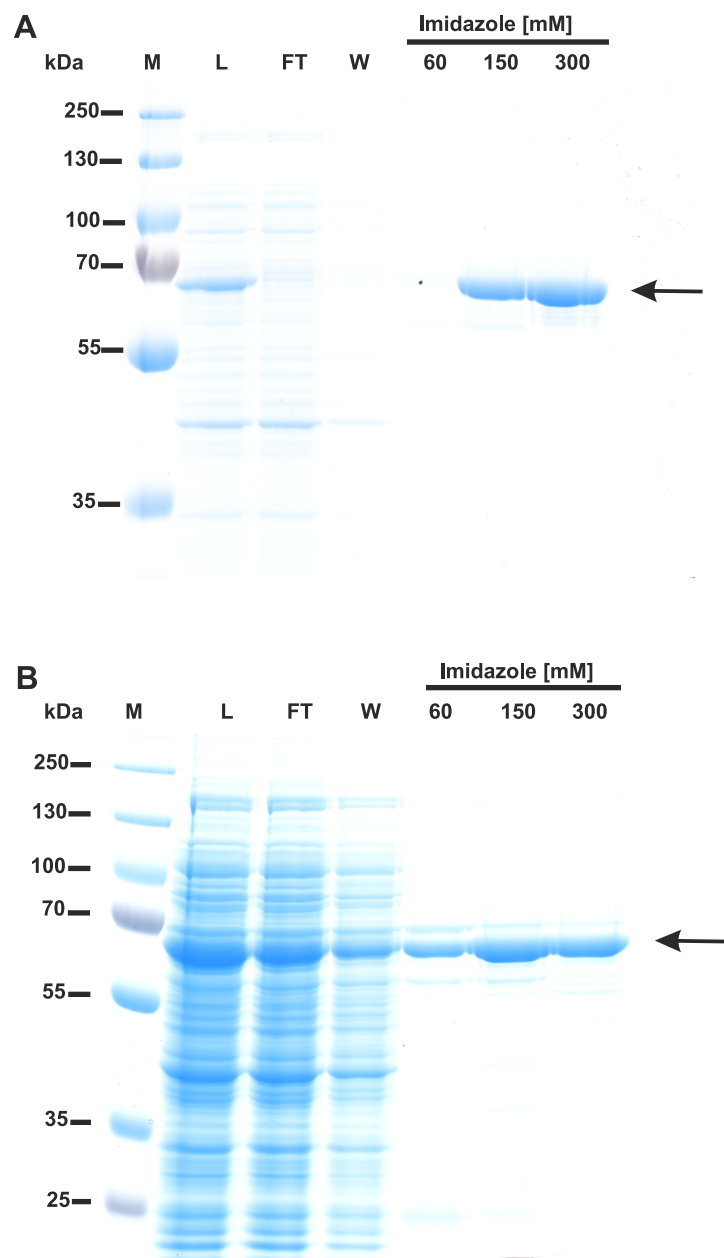

Supplement: Supplemental Information 1 — Recombinant human SETD3 protein (A) and its enzymatically inactive variant R253A (B) were purified by affinity chromatography using nickel-Sepharose (HisTrap FF crude), as detailed in the ‘Materials and Methods’ section. For SDS-PAGE analysis, 11 μ L of each eluted fraction was loaded onto a 12% polyacrylamide gel, electrophoresed, and the gel was stained with colloidal Coomassie Brilliant Blue. Arrows indicate protein bands corresponding to the purified SETD3 or R253A protein. M, prestained protein marker; L, cell-free lysate of E. coli loaded on the column; FT, flow through; W, wash; Fractions 60 to 300 were eluted with the indicated concentrations of imidazole. [file peerj-13-20195-s001.pdf]

Supplementary Figure 2

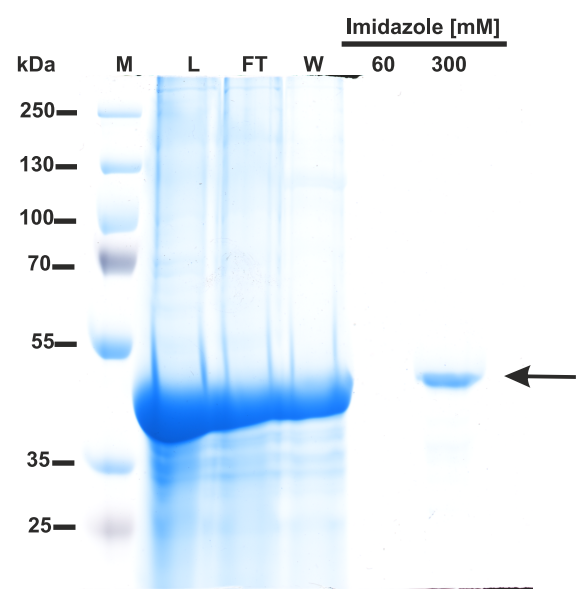

Supplement: Supplemental Information 2 — Recombinant human α-centractin (ACTR1A) was purified to homogeneity by affinity chromatography using nickel-Sepharose (HisTrap FF crude), as described in the ‘Materials and Methods’ section. For SDS-PAGE analysis, 15 μ L of each eluted fraction was loaded onto a 12% polyacrylamide gel. The proteins were separated by electrophoresis, and the gel was stained with colloidal Coomassie Brilliant Blue. The arrow indicates the protein band corresponding to the purified α-centractin. M, prestained protein marker; L, urea-washed inclusion bodies of E. coli loaded on the column; FT, flow through; W, wash; Fractions 60 and 300 were eluted with the indicated concentrations of imidazole. [file peerj-13-20195-s002.pdf]

# Supplementary Figure 3.

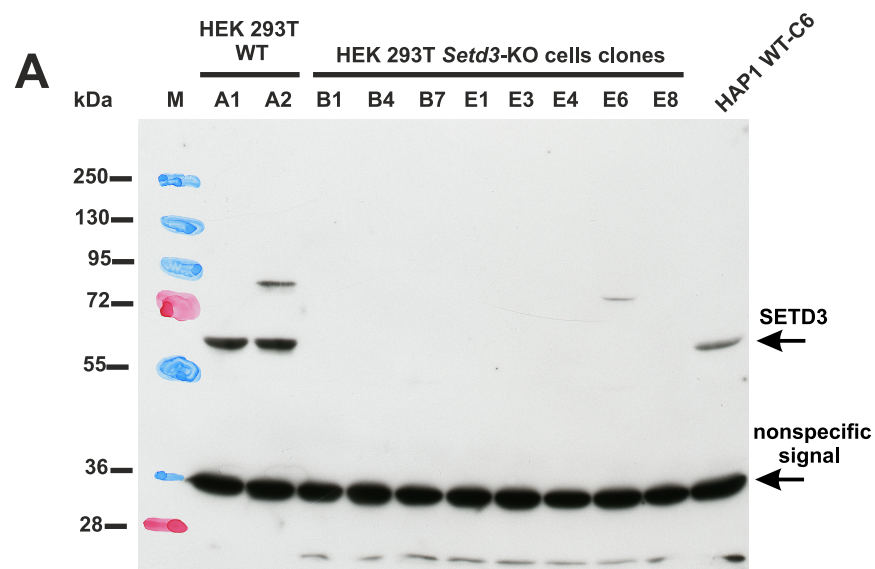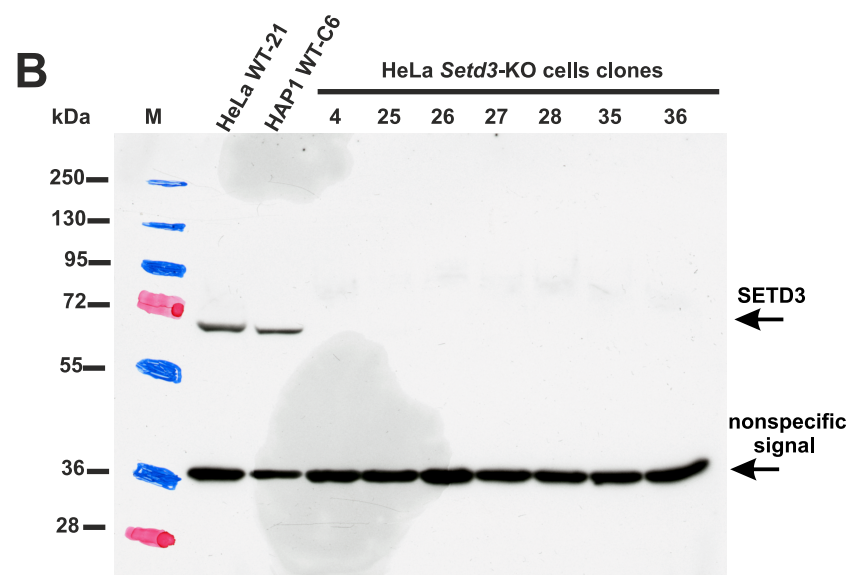

Supplement: Supplemental Information 3 — (A) SETD3 expression in SETD3-deficient HEK 293T cells. Western blot analysis was performed to compare SETD3 protein levels in eight clonal HEK 293T cell lines deficient in SETD3 with control cell lines: HEK293T wild-type (WT) cells (A1 and A2) and the HAP1 cell line (WT-C6). (B) SETD3 expression in SETD3-deficient HeLa cells. Western blot analysis was performed to compare SETD3 protein levels in seven clonal HeLa cell lines deficient in SETD3 with control cell lines: HeLa wild-type cells (WT-21) and the HAP1 cell line (WT-C6). The analyses were performed using 100 μ g of the cell lysate protein loaded per lane. Rabbit primary antibody against the human SETD3 (ab174662, Abcam) along with a horseradish-peroxidase-conjugated goat anti-rabbit secondary antibody were used for detection. Enhanced chemiluminescence (ECL) was used for visualization. Note: A non-specific band observed at approximately 36 kDa is consistent with the manufacturer’s specifications for the primary antibody. [file peerj-13-20195-s003.pdf]

Supplementary Figure 4.

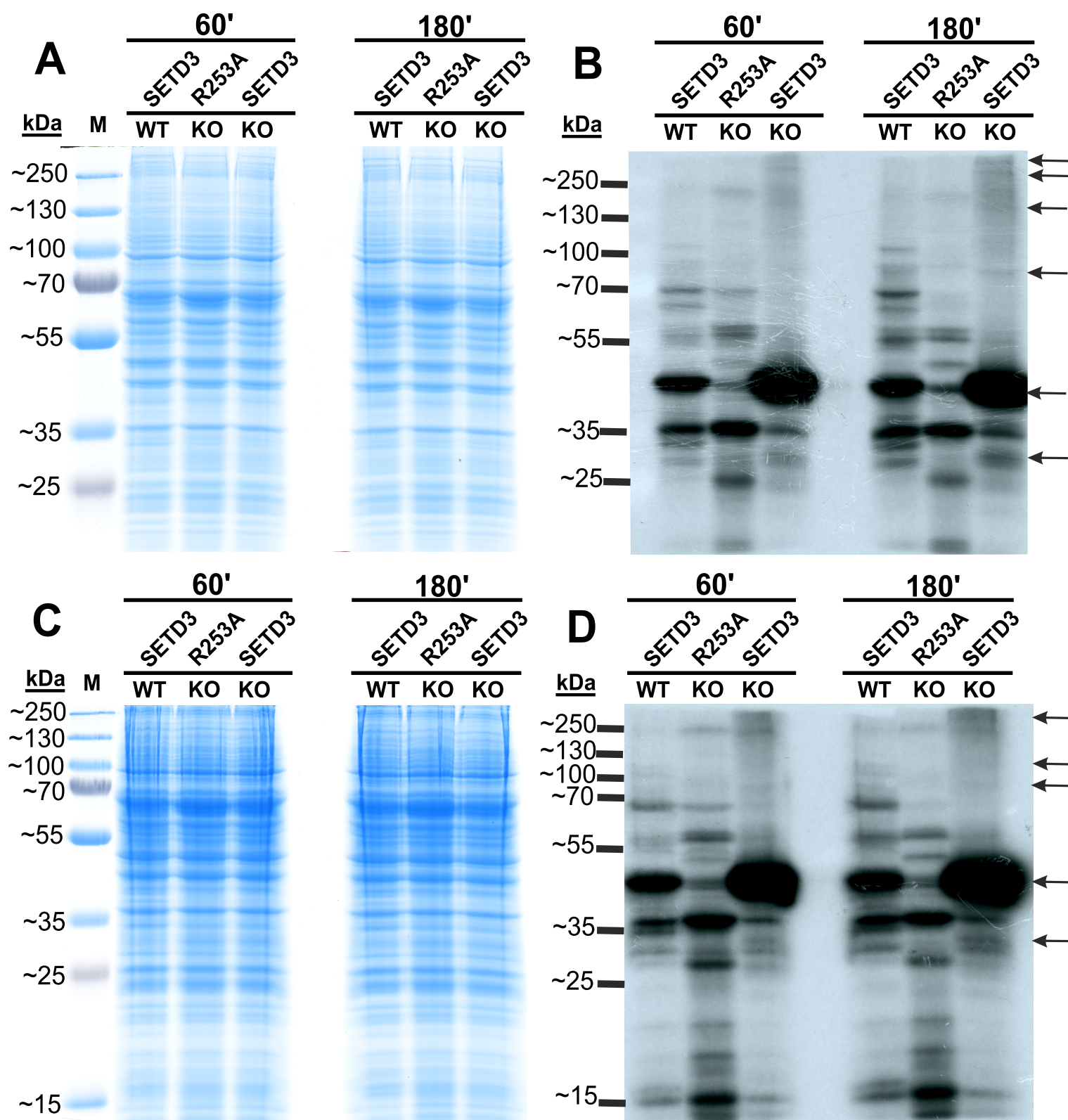

Supplement: Supplemental Information 4 — Cell-free lysates of human wild-type (WT) or SETD3 knockout (KO) HAP1 cells (230 μ g protein) were preincubated with [1 H]SAM for 30 min and then gel-filtered to remove [1H]SAM. Next, [3H]SAM (1x10^6^ cpm) was added to the reaction mixture and the radiolabeling of the protein was started by adding homogeneous recombinant SETD3 or its inactive R253A variant (10 μ g protein). The reaction mixtures were incubated for 60 or 180 min at 37 °C and resolved by SDS-PAGE (A, C). The [3H] methylated proteins were detected by fluorography for 4 weeks at −80 °C as described in Materials and Methods (B, D). Either 30 μ g (A, B) or 60 μ g (C, D) protein were loaded on the gels. The arrows show the methylated polypeptides that differentiate the lysates incubated with inactive (R253A) and active (SETD3) methyltransferase. M, prestained protein marker. [file peerj-13-20195-s004.pdf]

Supplementary Figure 5.

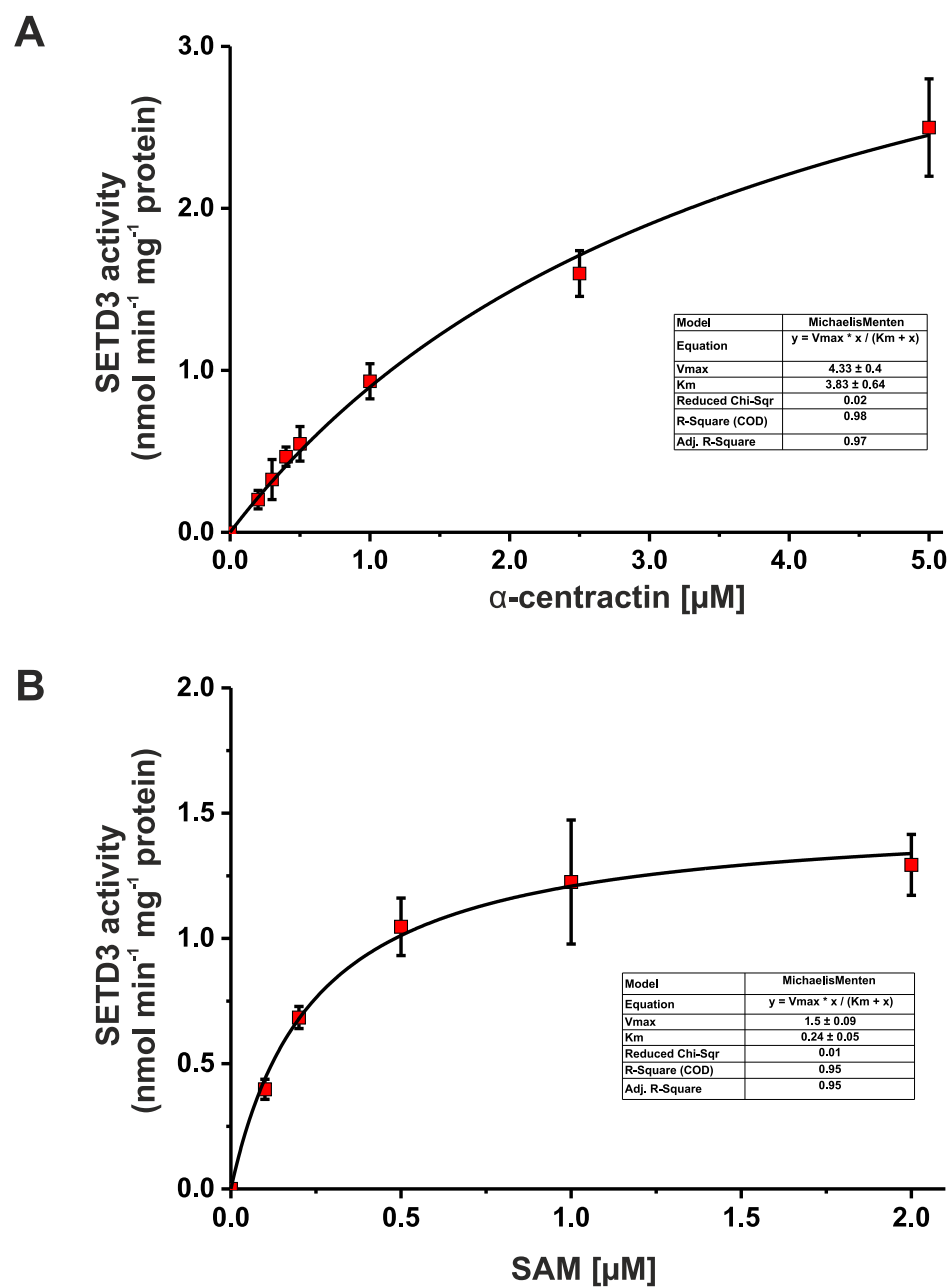

Supplement: Supplemental Information 5 — Michaelis-Menten plots for (A) α-centractin and (B) S-adenosyl-L-methionine (SAM) are shown. The activity of methyltransferase was followed radiochemically by measuring the incorporation of the [3 H]methyl group into α-centractin protein. Determinations for SAM were performed with SETD3 preparations (0.5 μ g protein, 140 nM) that were incubated for 5 min at 37 °C in the reaction mixture containing 4 μ M recombinant α-centractin and variable concentrations of [1H+3H] SAM (0.1–2.0 μ M, 330,000 cpm), while the measurements for α-centractin (0.2–5 μ M) were obtained following a 5-min incubation of SETD3 in the presence of 1 μ M concentration of [1H+3H] SAM (50 pmol, 330,000 cpm). In all experiments, the reaction mixture contained the homogenous recombinant SAH nucleosidase (0.8 μ g protein, 300 nM, E. coli) and adenine deaminase (2 μ g protein, 300 nM, B. subtilis) to prevent S-adenosyl-L-homocysteine (SAH) accumulation. Values are the means of three separate experiments. Values are the means ± SD (error bars) of three independent experiments. The curves were plotted employing Origin 2025 software (OriginLab, USA) and nonlinear regression analysis. [file peerj-13-20195-s005.pdf]
